# PM2.5 inhalation induces intracranial atherosclerosis which may be ameliorated by omega 3 fatty acids

**DOI:** 10.18632/oncotarget.23347

**Published:** 2017-12-16

**Authors:** Longfei Guan, Xiaokun Geng, Jiamei Shen, James Yip, Fengwu Li, Huishan Du, Zhili Ji, Yuchuan Ding

**Affiliations:** ^1^ China-America Institute of Neuroscience, Beijing Luhe Hospital, Capital Medical University, Tongzhou Qu, China; ^2^ Department of Neurosurgery, Wayne State University School of Medicine, Detroit, Michigan, USA; ^3^ Department of Neurology, Beijing Luhe Hospital, Capital Medical University, Tongzhou Qu, China; ^4^ Department of General Surgery, Beijing Luhe Hospital, Capital Medical University, Tongzhou Qu, China

**Keywords:** high-cholesterol diet (HCD), NG-nitro-L-arginine methyl ester (L-NAME), brain, inflammation, air pollution

## Abstract

**Background:**

Intracranial atherosclerosis (ICA) a major health problem. This study investigated whether inhalation of fine airborne particulate matters (PM2.5) causes ICA and whether omega-3 fatty acids (O3FA) attenuated the development of ICA.

**Results:**

Twelve but not 6 week exposure significantly increased triglycerides (TG) in normal chow diet (NCD), while PM2.5 enhanced all lipid profiles (TG, low density lipoprotein (LDL) and cholesterol (CHO)) after both 6 and 12-week exposure with high-cholesterol diet (HCD). PM2.5 exposure for 12 but not 6 weeks significantly induced middle cerebral artery (MCA) narrowing and thickening, in association with the enhanced expression of inflammatory cytokines, (interleukin 6 (IL-6), tumor necrosis factor alpha (TNF-α), monocyte chemoattractant protein-1 (MCP-1), interferon gamma (IFN-γ)), vascular cell adhesion molecule 1 (VCAM-1) and inducible nitric oxide synthase (iNOS). O3FA significantly attenuated vascular alterations, even without favorable changes in lipid profiles, in association with reduced expression of IL-6, TNF-α, MCP-1, IFN-γ, VCAM-1 and iNOS in brain vessels.

**Conclusions:**

PM2.5 exposure for 12 weeks aggravates ICA in a dietary model (HCD + short-term L-NAME), which may be mediated by vascular inflammation. O3FA dietary supplementation prevents ICA development and inflammatory reaction in cerebral vessels.

**Methods:**

Adult Sprague-Dawly rats were under filtered air (FA) or PM2.5 exposure with NCD or HCD for 6 or 12 weeks. Half of the HCD rats were treated with O3FA (5 mg/kg/day) by gavage. A total of 600 mg NG-nitro-L-arginine methyl ester (L-NAME, 3 mg/mL) per rat was administered over two weeks as supplementation in the HCD group. Blood lipids, including LDL, CHO, TG and high density lipoprotein (HDL), were measured at 6 and 12 weeks. ICA was determined by lumen diameter and thickness of the MCA. Inflammatory markers, IL-6, TNF-α, MCP-1, IFN-γ, VCAM-1 and iNOS were assessed by real-time PCR for mRNA and Western blot for protein expression.

## INTRODUCTION

Airborne fine particulate matter (aerodynamic diameter <2.5 µm, PM2.5), a component of air pollution, has been epidemiologically associated with respiratory and cardiovascular diseases [1–5]. Among them, atherosclerosis is one of the major public health concerns. Intracranial atherosclerosis (ICA), involving major cerebral arteries such as the internal carotid, middle cerebral, vertebral, and basilar arteries, is a highly prevalent cause of ischemic stroke [6–9]. The etiology of atherosclerosis is very complicated; the risk factors include genetic defects, smoking, hyperlipidemia, hypertension, lack of exercise, etc. A recent study in China demonstrated that elevated PM2.5 concentration was associated with first hospital admissions for ischemic stroke [10]. Moreover, epidemiological and experimental studies demonstrated that PM2.5 exposure contributes to the development of atherosclerosis [11–13].

PM2.5-induced inflammation is considered a key molecular mechanism of PM2.5-mediated toxicity [13]. Many studies indicated that the inflammatory response is also critical for the development of atherosclerosis [12, 13]. Sun *et al.* used whole body exposure to concentrate ambient PM2.5 and demonstrated that long-term exposure to PM2.5 can potentiate plaque development and vascular inflammation in apoE-deficient mice [13, 14]. The atherosclerotic plaque development consists of lesion initiation, foam cell formation and fibrous plaque formation [15], all of which have been well-demonstrated by animal studies [23]. Endothelial dysfunction is also recognized as the crucial step in atherogenesis. Many studies have confirmed the involvement of various inflammatory mediators in the initial proatherogenic processes, such as the upregulation of adhesion molecules on endothelial cells, binding of low density lipoproteins to the endothelium, activation of macrophages and proliferation of vascular smooth muscle cells [16].

Numerous epidemiology studies and clinical trials indicated that omega-3 fatty acids (O3FA) prevent atherosclerotic disease development in humans [17–19]. Studies of Mediterranean populations showed that regular consumption of dietary O3FA is associated with a lower incidence of cardiovascular disease [20]. A recent cohort study showed that higher circulating levels of docosahexaenoic acid (DHA) were inversely associated with the incidence of atherothrombotic stroke and docosapentaenoic acid (DPA) with cardioembolic stroke [21]. Among the therapeutic modalities for cardiovascular atherosclerosis, the effects of O3FA have received considerable attention, but their efficacy as secondary prevention remains controversial [22, 23]. Despite the vast majority of studies attempting to elucidate O3FA’s mechanistic effects on atherosclerosis, very few studies on O3FA and its effects on intracranial atherosclerotic stenosis (ICAS) have been carried out [20, 24, 25].

Recently, our group effectively developed a rat model of intracranial atherosclerosis by high-cholesterol diet (HCD) and NG-nitro-L-arginine methyl ester (L-NAME, 3 mg/mL) administration. This clinically-relevant model would be beneficial for studying ICAS [26]. In this work, we focused on the impact of “real-world” PM2.5 exposure and O3FA supplement on ICA and explored the underlying mechanism.

## RESULTS

### PM2.5 concentrations in “real world”

**Figure 1:**
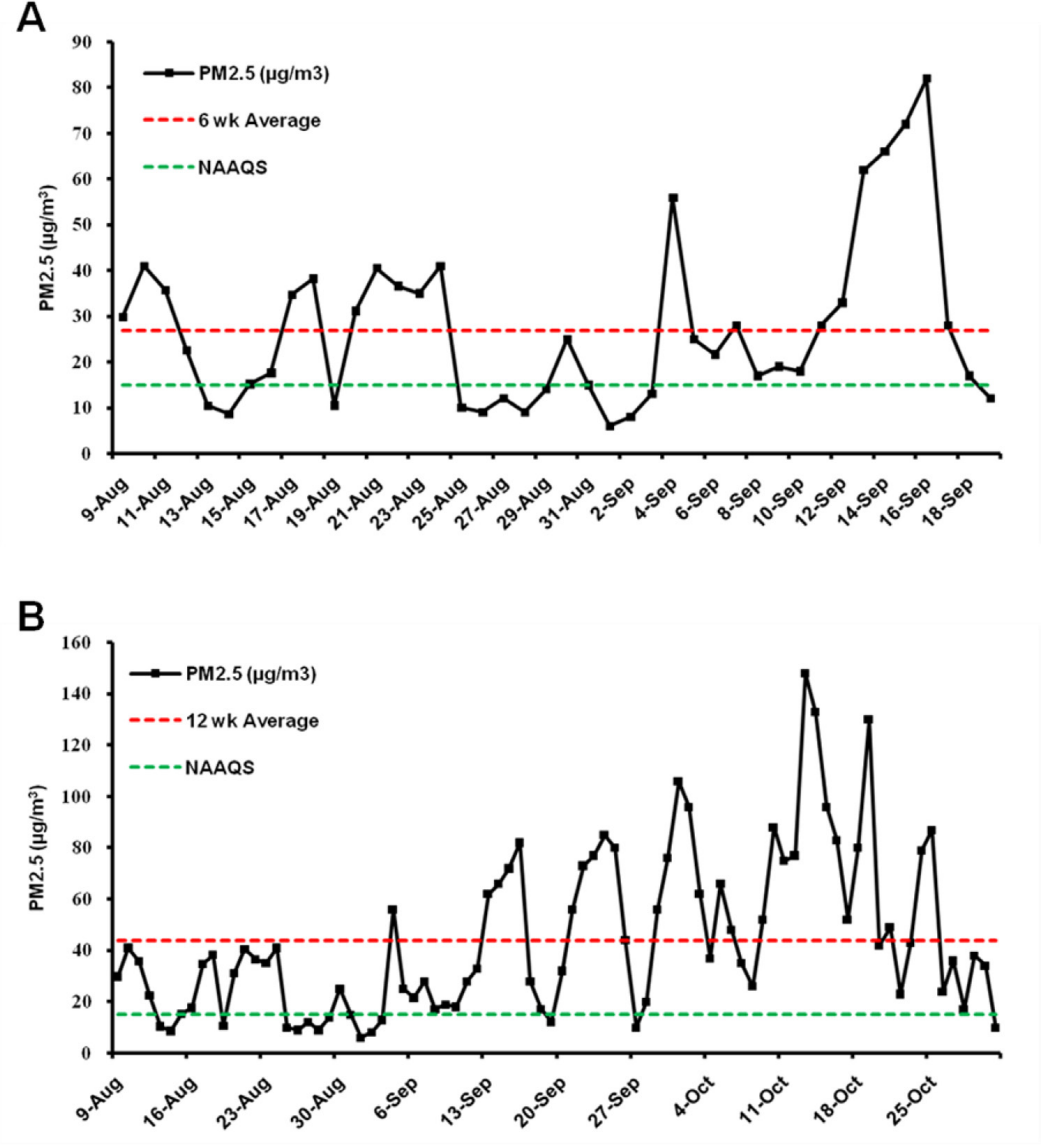
PM2.5 concentration during the exposure periods The mean daily PM2.5 concentration during the 6 week (**A**) and 12 week (**B**) exposure is displayed. The red dotted line indicates the average PM2.5 concentration during the exposure period and the green dotted line indicates the annual average PM2.5 National Ambient Air Quality Standard (NAAQS) of 15 µg/m^3^ in China. The mean daily PM2.5 concentration during the 6 week and 12 week exposure was 27 µg/m^3^ and 44 µg/m^3^, respectively (as the red dotted line indicated).

Animals were exposed to either ambient PM2.5 or FA for 24 h/day, 7 days/week for 6 (from August 9, 2016 to September 19, 2016) or 12 weeks (from August 9, 2016 to October 31, 2016) in a “real-world” airborne PM2.5 exposure system in Tongzhou District, Beijing, China [27]. During exposure, the mean daily concentration of ambient PM2.5 was monitored using an individual particle monitor (pDR1500, Thermo, USA). The average daily concentration variation of PM2.5 is displayed in Figure 1. The mean daily PM2.5 concentration during the 6-week exposure was 27 µg/m^3^ (the red dotted line in Figure 1A), while the PM2.5 concentration for the 12 week exposure was 44 µg/m^3^ (Figure 1B), which is approximately 3-fold higher than the 15 µg/m^3^ annual average PM2.5 National Ambient Air Quality Standard (NAAQS) in China and 4.4-fold higher than the 10 µg/m^3^ annual average PM2.5 WHO Air Quality Guidelines [4].

### Effects of PM2.5 exposure on blood lipids

**Figure 2:**
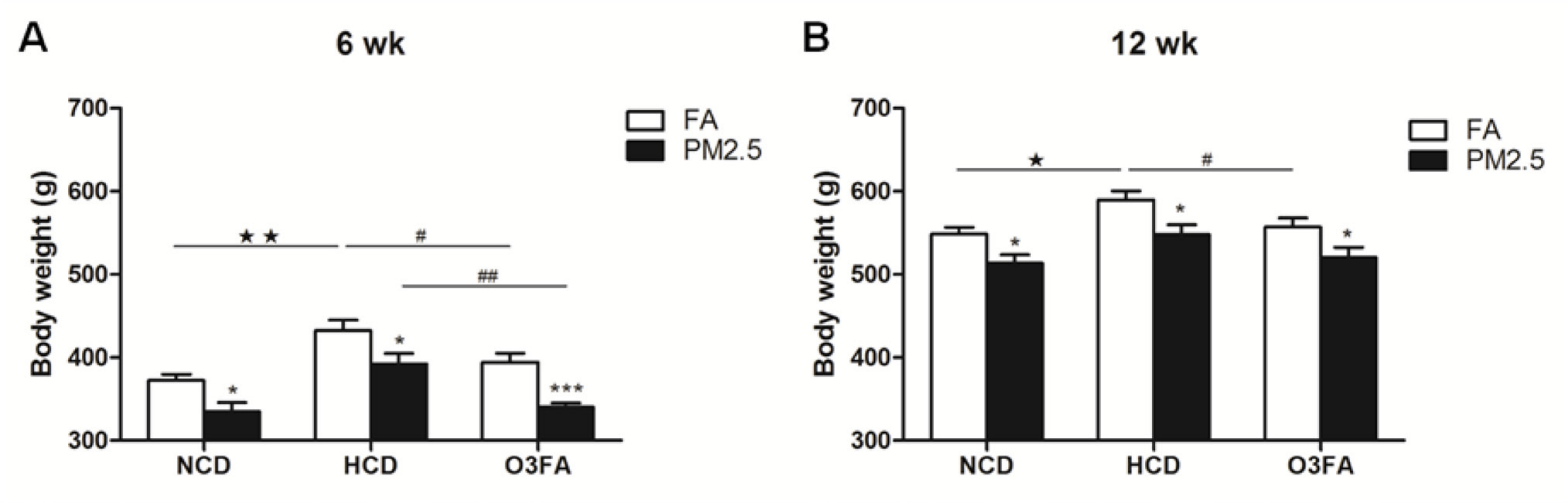
Body weight changes of rats during the exposure period Body weight in the normal chow diet (NCD), high-cholesterol diet (HCD) and O3FA group after 6 weeks (**A**) and 12 weeks (**B**) of PM2.5 exposure is shown. Data are presented as mean ± SE; *n* = 9 rats/group. ^*^p < 0.05, ^***^p < 0.001 compared to the filtered air group; ^★^*p* < 0.05, ^★★^*p* < 0.01 compared to the normal chow diet group; ^#^*p* < 0.05, ^##^*p* < 0.01 as compared to the high-cholesterol diet group.

**Figure 3:**
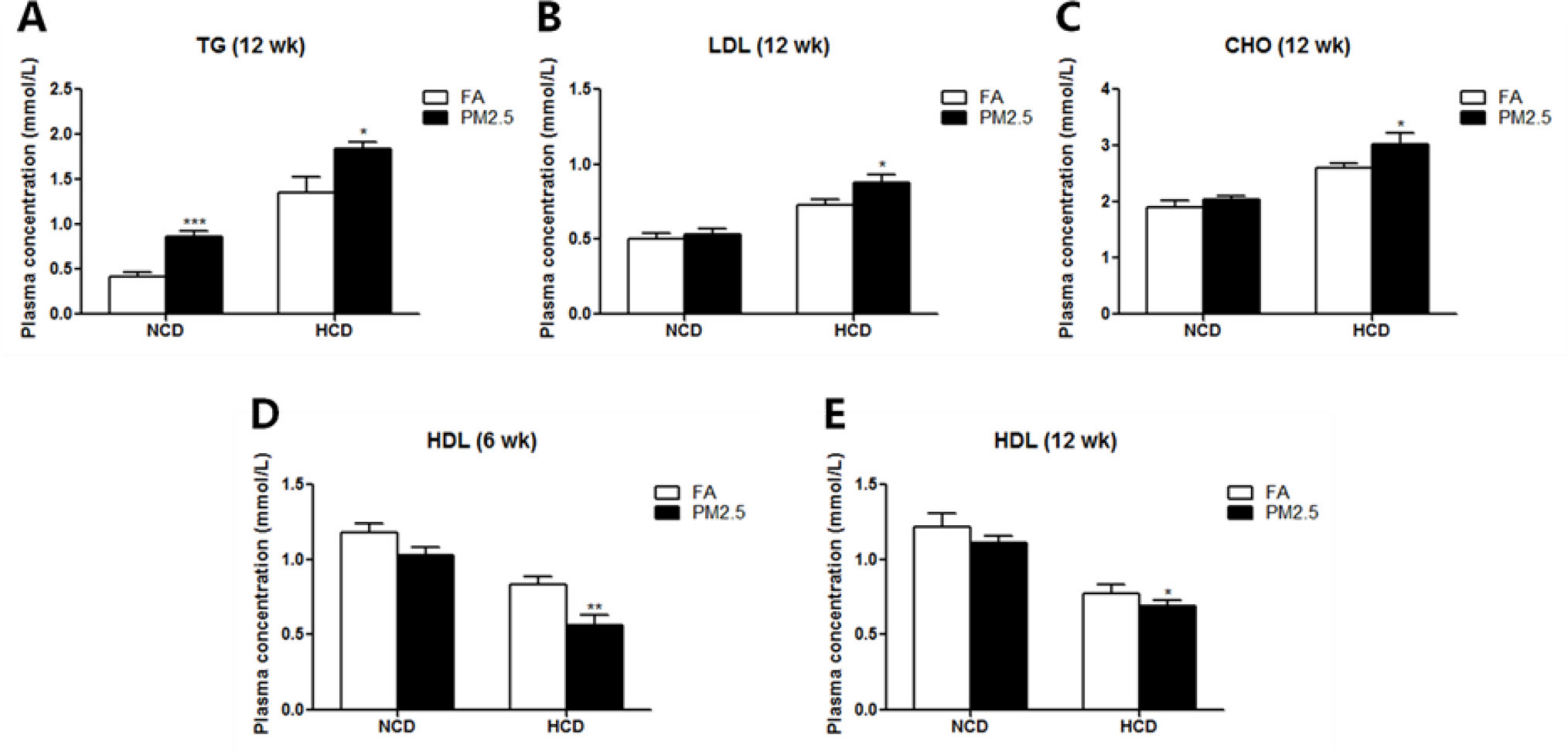
Effects of PM2.5 exposure on blood lipids TG (**A**), LDL (**B**), CHO (**C**) levels after 12 weeks of PM2.5 exposure, and HDL level after 6 (**D**) or 12 (**E**) weeks of PM2.5 exposure are shown. The level of TG was significantly elevated after 12 weeks of PM2.5 exposure as compared to the FA group in both normal chow diet and high-cholesterol diet group (A). Twelve weeks of PM2.5 exposure significantly elevated the levels of LDL and CHO in the high-cholesterol diet group (B, C). Both 6 and 12 weeks of PM2.5 exposure decreased the level of HDL in the high-cholesterol diet group (D, E). Values are mean ± SE (*n* = 9). ^*^p < 0.05, ^**^p < 0.01 and ^***^p < 0.001 as compared to the FA group.

During the exposure, body weight was recorded every week. As shown in Figure 2, PM2.5 exposure for 6 or 12 weeks significantly decreased body weight in either diet (Figure 2A, 2B). HCD significantly increased body weight compared to normal chow diet, while O3FA decreased body weight, especially under PM2.5. As shown in Table 1, both 6 and 12 weeks of HCD significantly elevated the levels of triglycerides (TG), cholesterol (CHO) and low-density lipoprotein (LDL), and decreased high-density lipoprotein (HDL) level in both FA and PM2.5 group. Meanwhile, O3FA significantly ameliorated TG and CHO elevation in 6 weeks and TG, CHO and LDL in 12 weeks. PM2.5 exposure for 6 weeks, however, did not alter plasma lipids in either diet, except HDL in the HCD group (Figure 3D). With the extension of exposure time, compared to the FA group, PM2.5 exposure for 12 weeks significantly increased TG, CHO and LDL levels, and decreased HDL level, especially in the HCD group (Figure 3A–3C, 3E).

**Table 1:** Effects of HCD and O3FA on blood lipids

Blood lipids (mmol/L)		FA			PM2.5		
		NCD	HCD	O3FA	NCD	HCD	O3FA
6 week (Mean ± SEM)	TG	0.51 ± 0.05	1.10 ± 0.18^★★^	0.63 ± 0.08^#^	0.52 ± 0.11	1.20 ± 0.19^★★^	0.65 ± 0.09^#^
	LDL	0.52 ± 0.02	0.72 ± 0.04^★^	0.54 ± 0.06^#^	0.59 ± 0.03	0.78 ± 0.03^★^	0.65 ± 0.03^#^
	CHO	1.50 ± 0.11	2.04 ± 0.15^★^	1.71 ± 0.21	1.78 ± 0.10	2.40 ± 0.09^★★^	2.09 ± 0.17
	HDL	1.18 ± 0.06	0.84 ± 0.05^★★^	1.02 ± 0.08	1.03 ± 0.05	0.56 ± 0.07^★★^	0.51 ± 0.05
12 week (Mean ± SEM)	TG	0.41 ± 0.05	1.35 ± 0.17^★★^	0.78 ± 0.09^##^	0.87 ± 0.07	1.83 ± 0.09^★★^	0.87 ± 0.07^##^
	LDL	0.50 ± 0.04	0.73 ± 0.04^★★^	0.41 ± 0.05^##^	0.53 ± 0.04	0.88 ± 0.05^★★^	0.63 ± 0.04^##^
	CHO	1.91 ± 0.12	2.60 ± 0.08^★★^	2.08 ± 0.17^#^	2.05 ± 0.06	3.03 ± 0.21^★★^	2.25 ± 0.15^##^
	HDL	1.22 ± 0.09	0.77 ± 0.06^★★^	0.81 ± 0.06	1.11 ± 0.05	0.69 ± 0.05^★★^	0.82 ± 0.04

Values are mean ± SE (*n* = 9). ^★^*p* < 0.05 and ^★★^*p* < 0.01 compared to the normal chow diet (NCD) group; ^#^*p* < 0.05 and ^##^*p* < 0.01 as compared to the high-cholesterol diet (HCD) group. O3FA: Omega-3 fatty acids.

### Effects of PM2.5 on MCA morphology

**Figure 4:**
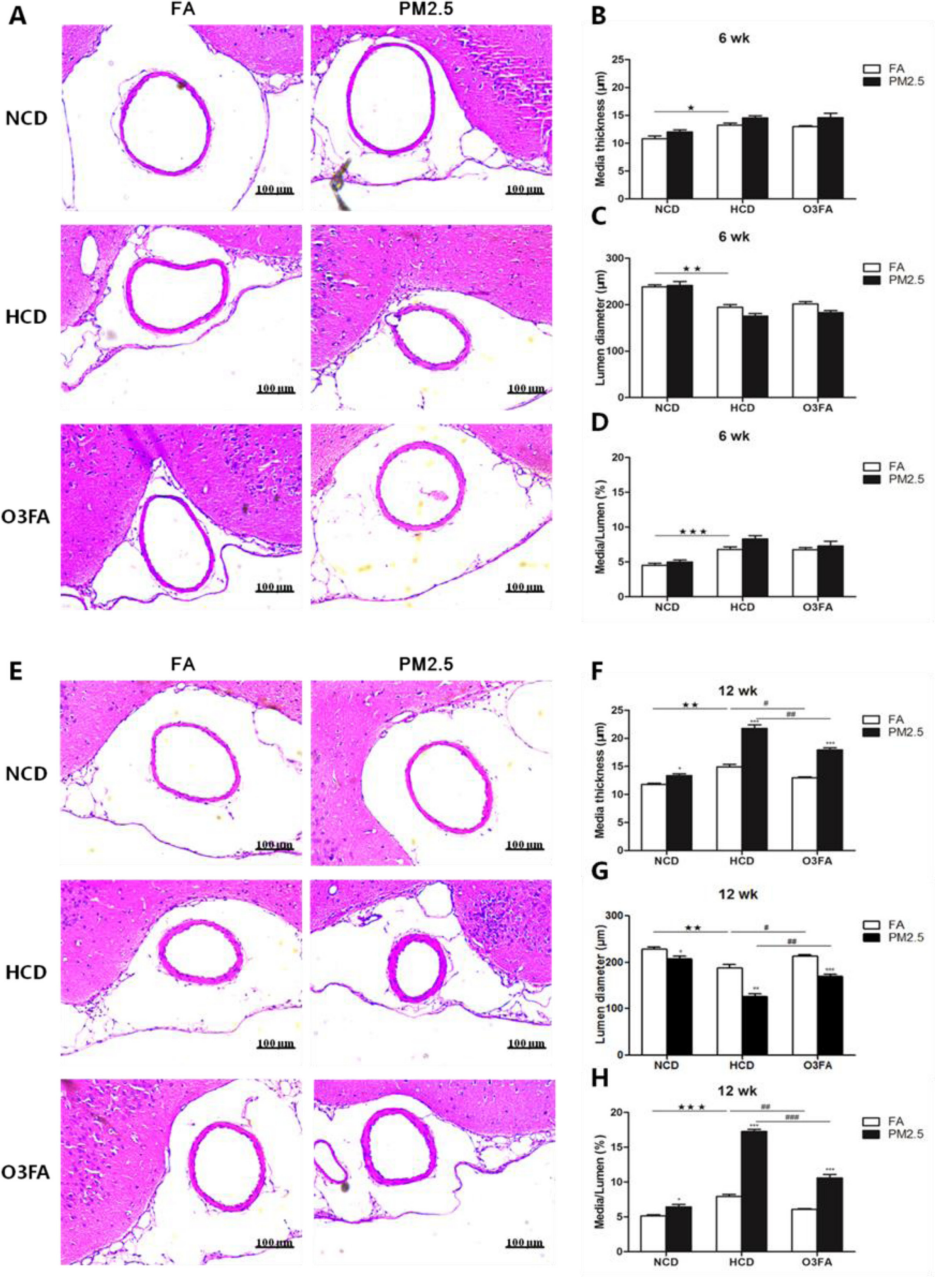
Representative photomicrographs of hematoxylin-eosin staining of middle cerebral artery sections MCA media thickness and lumen diameter as compared to the filtered air group after 6 week (**A**) or 12 week exposure (**E**). Quantitative analyses show media thickness (µm), lumen diameter (µm) and media-lumen ratio (%) (**B**–**D**) represents 6 week PM2.5 exposure and (**F**–**H**) represents 12 week PM2.5 exposure values). (*n* = 9 for each group). Values are represented as mean ± SE.^*^p < 0.05, ^**^p < 0.01 and ^***^p < 0.001 as compared to the FA group; ^★^*p* < 0.05, ^★★^*p* < 0.01 and ^★★★^*p* < 0.001 as compared to the normal chow diet group; ^#^*p* < 0.05, ^##^*p* < 0.01 and ^###^*p* < 0.001 as compared to the high-cholesterol diet group.

To determine the severity of atherosclerosis, middle cerebral artery (MCA) media thickness and lumen diameter were measured. Figure 4A–4D demonstrates that 6 weeks of PM2.5 exposure did not change the MCA media thickness and lumen diameter as compared to the FA group, but 6 weeks of HCD did significantly increase the media thickness and decrease the lumen diameter, consistent with our previous study [28]. After 12 weeks of exposure, MCA thickness in the PM2.5 group with normal chow diet (NCD) (13.4 ± 0.6 µm) or HCD (21.8 ± 0.8 µm) clearly showed a significant increase when compared to the FA group (NCD for 11.8 ± 0.4 µm and HCD for 14.9 ± 0.8 µm) (Figure 4E, 4F). In addition, the MCA lumen diameter of the PM2.5 group (NCD for 208.0 ± 14.8 µm and HCD for 126.5 ± 10.5 µm) significantly decreased as compared to the FA group (NCD for 228.9 ± 7.6 µm and HCD for 188.4 ± 11.5 µm) (Figure 4E, 4G). Figure 4D and 4H show the ratio of media thickness and lumen diameter, further suggesting the development of ICA. Consistently, O3FA significantly attenuated the decreased MCA lumen diameter and increased media thickness, especially after 12 weeks of exposure. As for the effects of diet, HCD decreased lumen diameter, increased MCA media thickness and increased media-lumen ratio as compared to NCD.

### Effects of PM2.5 on vascular inflammation in the brain

**Figure 5:**
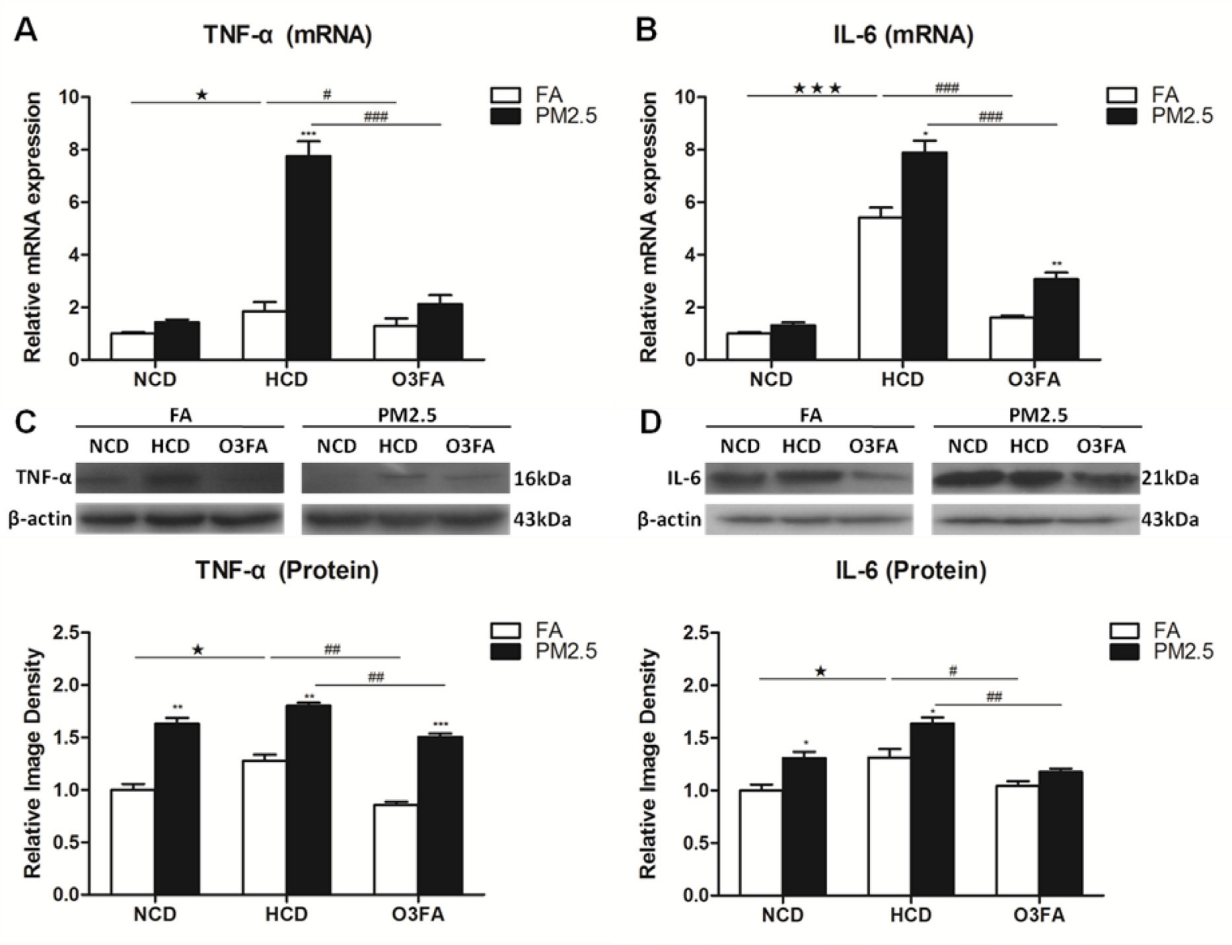
Effects of PM2.5 exposure on the expression of TNF-α and IL-6 in brain microvessels The mRNA expression of TNF-α (**A**) and IL-6 (**B**), and protein levels of TNF-α (**C**) and IL-6 (**D**) after 12 week PM2.5 exposure were detected by Real-time PCR and Western blot analyses, respectively. Data are presented as mean ± SE (*n* = 6). ^*^p < 0.05, ^**^p < 0.01 and ^***^p < 0.001 as compared to the FA group; ^★^*p* < 0.05, ^★★★^*p* < 0.001 as compared to the normal chow diet group; ^#^*p* < 0.05, ^##^*p* < 0.01 and ^###^*p* < 0.001 as compared to the high-cholesterol diet group.

**Figure 6:**
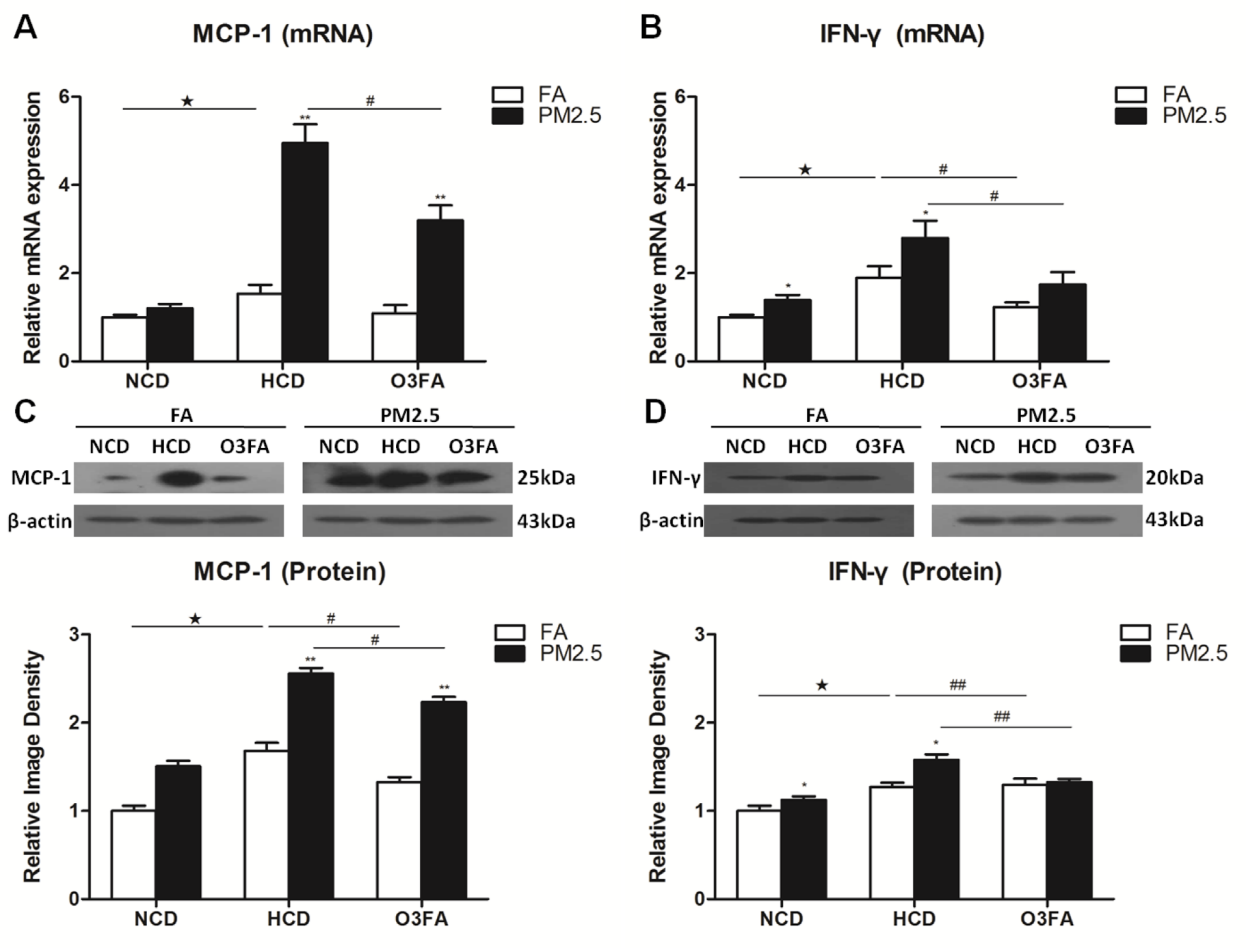
Effects of PM2.5 exposure on the expression of MCP-1 and IFN-γ in brain microvessels The mRNA expression of MCP-1 (**A**) and IFN-γ (**B**), and protein levels of MCP-1 (**C**) and IFN-γ (**D**) after 12 week PM2.5 exposure were detected by Real-time PCR and Western blot analyses, respectively. Values are represented as mean ± SE (*n* = 6). ^*^p < 0.05, ^**^p < 0.01 as compared to the FA group; ^★^*p* < 0.05 as compared to the normal chow diet group; ^#^*p* < 0.05, ^##^*p* < 0.01 as compared to the high-cholesterol diet group.

To determine the molecular basis underlying the effects of PM2.5 exposure on ICA, the expression of inflammation cytokines were examined in brain microvessels only after 12 weeks of PM2.5 exposure since the 6-week exposure did not change the brain MCA media. HCD significantly increased mRNA expression of the inflammatory cytokines TNF-α and IL-6 as compared to NCD, and O3FA significantly reversed the elevated expression (Figure 5A and 5B). Western blot analyses in Figure 5C and 5D displayed the same trend as its mRNA expression. Among the high cholesterol group, PM2.5 further increased mRNA and protein expression of TNF-α and IL-6. Messenger RNA expression of MCP-1 (Figure 6A) was increased by 54% (*p* < 0.05) after 12 weeks of HCD, and PM2.5 exposure further enhanced MCP-1 mRNA expression to 495% (*p* < 0.01). HCD also significantly increased mRNA expression of IFN-γ as compared to the normal chow diet group (Figure 6B). Western blot analyses in Figure 6C and 6D displayed the same trend as its mRNA expression. PM2.5 exposure further significantly increased MCP-1 protein (Figure 6C) and IFN-γ levels with HCD (Figure 6D). Treatment with O3FA significantly attenuated MCP-1 and IFN-γ up-regulation at both mRNA and protein levels.

### Effects of PM2.5 exposure on VCAM-1 and iNOS

**Figure 7:**
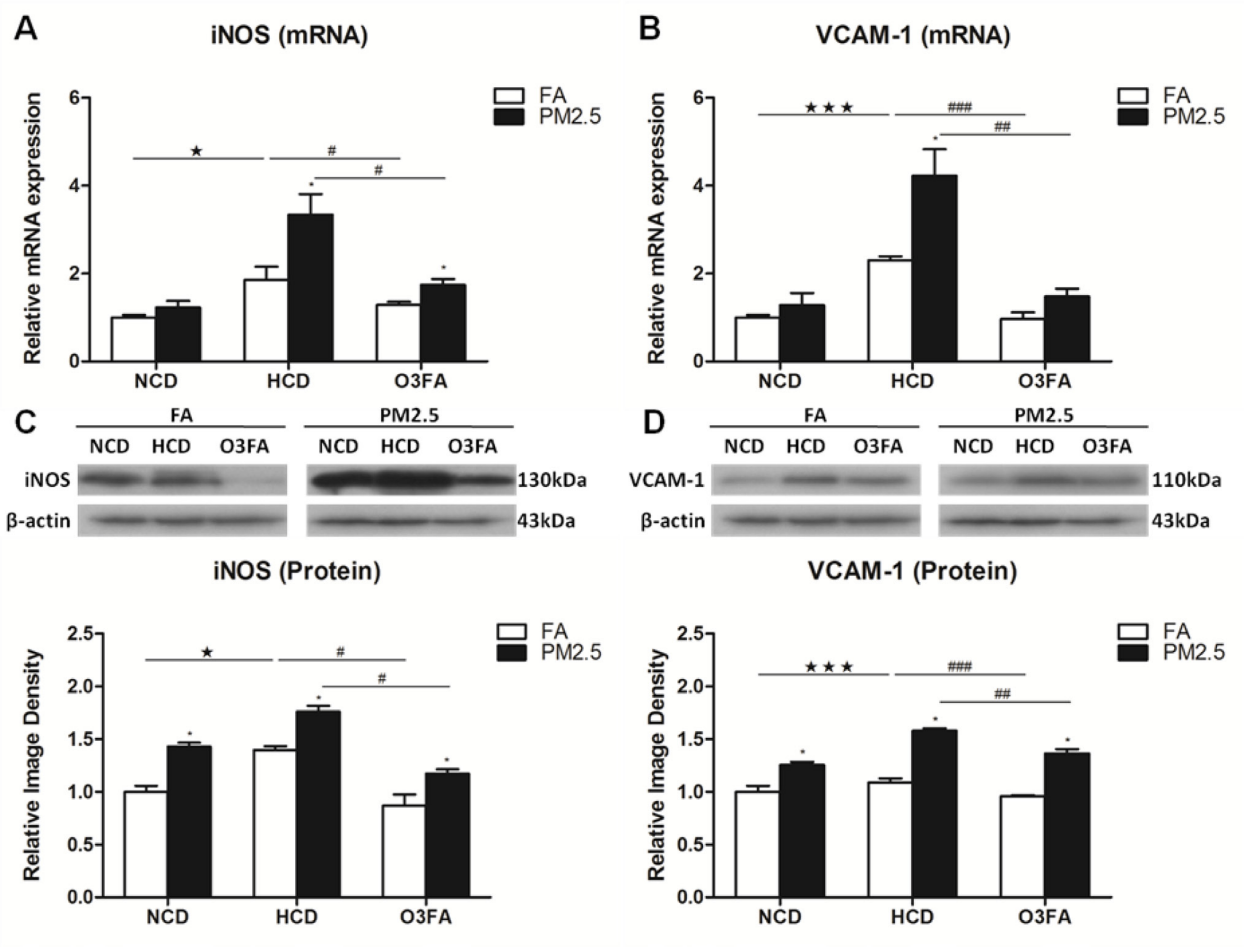
Effects of PM2.5 exposure on the expression of VCAM-1 and iNOS in brain microvessels The mRNA expression of iNOS (**A**) and VCAM-1 (**B**) and protein levels of iNOS (**C**) and VCAM-1 (**D**) after 12 week PM2.5 exposure were detected by Real-time PCR and Western blot analyses, respectively. Values are represented as mean ± SE (*n* = 6). ^*^p < 0.05 as compared to the FA group; ^★^*p* < 0.05, ^★★★^*p* < 0.001 as compared to the normal chow diet group; ^#^*p* < 0.05, ^##^*p* < 0.01 and ^###^*p* < 0.001 as compared to the high-cholesterol diet group.

We further investigated the effect of PM2.5 exposure on the expression of VCAM-1 and iNOS. We first found that HCD significantly increased the mRNA expression of iNOS (Figure 7A) and VCAM-1 (Figure 7B) as compared to NCD. Again, O3FA significantly decreased the mRNA expression of iNOS (Figure 7A) and VCAM-1 (Figure 7B). Western blot analyses in Figure 7C and 7D displayed the same trend in protein expression as its mRNA expression. Similarly, PM2.5 exposure further increased mRNA and protein expression of both iNOS and VCAM-1 with HCD. Treatment with O3FA significantly reversed the increased levels of mRNA and protein expression of iNOS and VCAM-1.

Taken together, these results indicate that PM2.5 exposure can induce brain vascular endothelium dysfunction, especially under HCD, whereas O3FA can ameliorate the endothelium dysfunction.

## DISCUSSION

**Figure 8:**
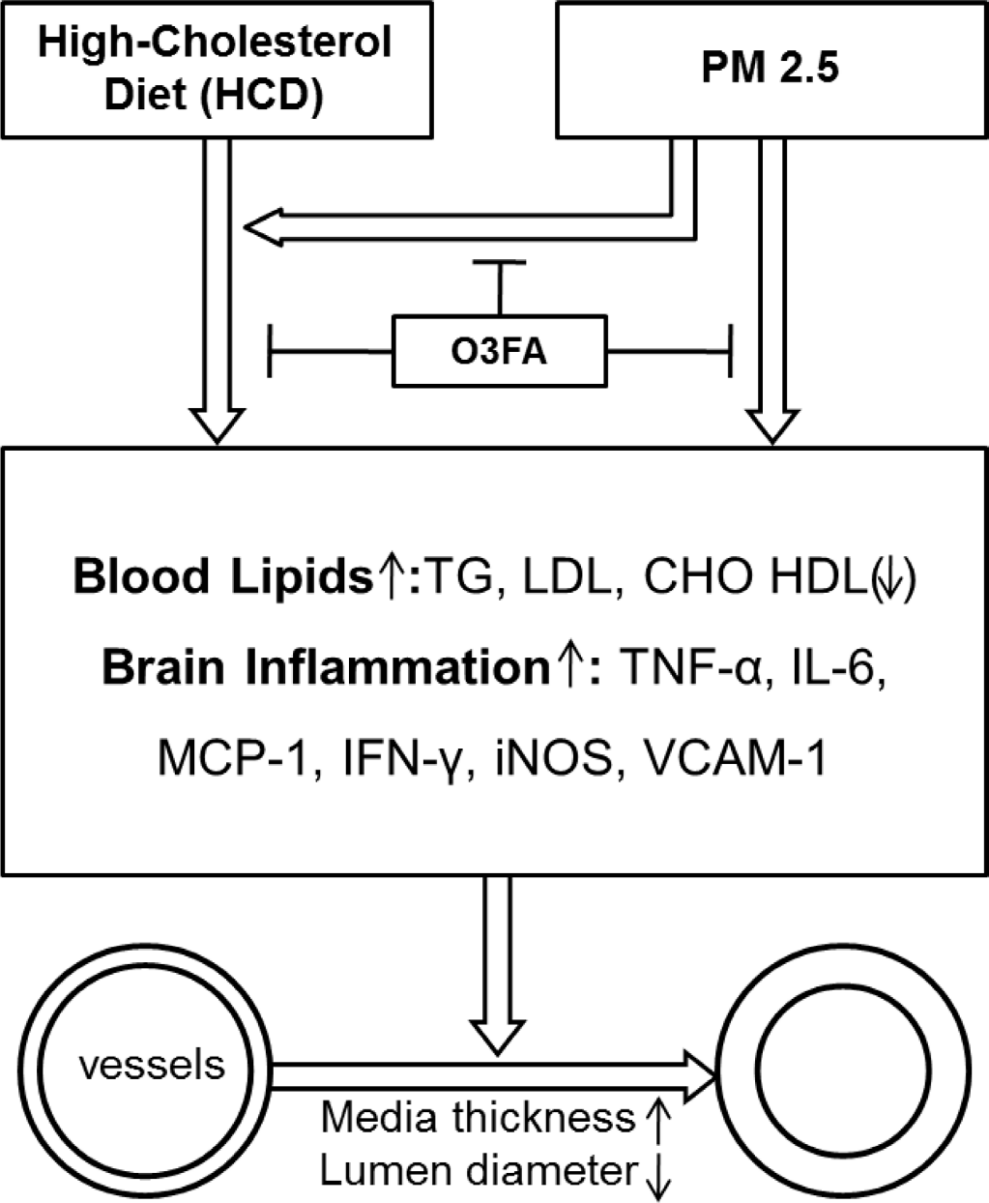
A schematic diagram depicting Intracranial Atherosclerosis induced by PM2.5 in a high-cholesterol diet rat model Blood lipids are induced by HCD, PM2.5 exposure or their combination, as evidenced by elevations in blood TG, LDL and CHO, with reduction in HDL expression. Subsequently, brain inflammation is also induced with elevations in TNF-α, IL-6, MCP-1, INF-ɣ, iNOS and VCAM-1. The resulting consequence is the development of intracranial atherosclerosis, with increased media thickness and narrowing of lumen diameter. The effects of HCD and PM2.5 can be attenuated with O3FA supplementation.

In the present study, we used a “real-world” exposure system to perform ambient inhalation exposure of experimental animals to environmental PM2.5. To our knowledge, this is the first study to investigate the effects of real-world PM2.5 exposure on ICA. Our major findings include: 1) Inhalation exposure to PM2.5 for 12 weeks (not 6 weeks) can induce ICA as evidenced by MCA thickening and MCA lumen narrowing; 2) HCD induced ICA in rats, and PM2.5 enhanced the HCD-induced atherosclerosis; 3) PM2.5-induced vascular inflammation (the elevated expression of TNF-α, IL-6, MCP-1 and IFN-γ) and vascular dysfunction (the increased expression of VCAM-1 and iNOS) may be the underlying mechanisms for atherosclerosis; 4) Oral supplementation of O3FA for 12 weeks ameliorated the detrimental effects of PM2.5 (as shown in Figure 8). These findings not only have important implications on the understanding of the complex effects of airborne PM2.5 pollution on ICA, but also provide a promising preventative strategy for ICAS by oral supplementation of the inexpensive, widely available O3FA.

Epidemiological studies have found an association between PM2.5 exposure and hospital admissions for stroke [10, 29]. Both *in vivo* and *in vitro* studies demonstrated the pro-atherosclerotic properties of PM2.5 exposure and reveal underlying mechanistic pathways [13, 30]. Atherosclerosis is a progressive disease characterized by the accumulation of lipids and fibrous plaques in the arteries. ICA is responsible for a significant number of strokes [31] and notably carries a high risk for recurrent stroke [32]. Currently, research using animal models of atherosclerosis have mainly focused on the vasculature around the heart, but very few study has been conducted intracranially [33, 34]. Given the lack of studies on ICA, we recently developed a new and complex intracranial atherosclerosis model in rats that consistently alters the morphometry of the major cerebral arteries by combining 6 weeks of HCD and an initial concurrent 2 weeks of L-NAME treatment [26]. In atherosclerosis research, the establishment of consistent and reliable methods for the elucidation of molecular mechanisms and testing of drugs for therapeutic efficiency is very important [35].

Previous studies have demonstrated that PM2.5 is associated with the development of systemic diseases, such as atherosclerosis [13]. *In vitro* studies typically used PM2.5 particles collected from the air to expose to cultured cells, thus mimicking PM2.5’s effects on human health [30, 36]. For animal studies, intranasal instillation or tracheal drip was often used as an *in vivo* exposure mode to study PM2.5’s effects [37, 38]. These exposure methods may determine the underlying molecular mechanisms by which PM2.5 may play a role on diseases. However, they do not reflect the effects of PM2.5 exposure in real-world. Considering this drawback, Sun *et al.* used a versatile aerosol concentration enrichment system to mimic the inhalation exposure of PM2.5 to animals. Using more physiologically-relevant whole body exposure to concentrated ambient PM2.5, Sun *et al.* and others demonstrated that long-term exposure to PM2.5 can potentiate plaque development and vascular inflammation in apoE-deficient mice [13, 14]. Due to the low PM2.5 concentrations at the study site, the mice were exposed to concentrated ambient PM2.5 at nominal 10× ambient concentrations. In the present study, we further used a “real-world” PM2.5 exposure system which was modified from the “versatile aerosol concentration enrichment system” (VACES) in the Sun *et al.* study [13]. Thus, the results can more accurately reflect the physiological significance of whole body exposure.

Many studies demonstrated that atherosclerosis is an inflammatory disease and that inflammation plays a pivotal role in the development of atherosclerosis [39]. Recent epidemiological and experimental studies demonstrated that PM2.5 air pollution is a risk factor that contributes to the development of atherosclerosis [11–13]. Pro-inflammatory cytokines released from alveolar macrophages (e.g. TNF-α, IL-6 and IFN-γ) can induce systemic inflammation and activate vascular inflammation. Blockade of inflammatory pathways has been shown to attenuate atherosclerosis after PM2.5 exposure. Studies on TLR4-deficient mice showed reduced vascular constriction and normal cytokine profile after chronic PM2.5 exposure [40]. Atherosclerosis is primarily triggered in response to activation of the arterial endothelium which stimulates the release of monocyte and T-lymphocyte-attracting chemokines, such as MCP-1 and IFN-γ, expressed highly in atherosclerotic regions and exhibit atherogenic actions [41].

VCAM-1, an important cell adhesion molecule, is a member of the immunoglobulin superfamily of proteins and crucially mediates the adhesion of lymphocytes, monocytes, eosinophils, and basophils to the vascular endothelium [42, 43]. Studies have shown that VCAM-1 plays a dominant role in the initiation of atherosclerosis [44]. Nitric oxide synthases (NOS) are a family of isoforms responsible for the synthesis of the potent dilator nitric oxide (NO). Expression of inducible NOS (iNOS) occurs in conditions of inflammation and produces large amounts of NO. In pathological conditions, iNOS is regarded as a harmful enzyme and is proposed to be a major contributor to diseases of the cardiovascular system such as atherosclerosis [45]. Our previous *in vitro* study suggests that PM2.5-induced ROS may function as signaling molecules, triggering VCAM-1 expression and further promoting monocyte adhesion to endothelial cells [30]. A previous study detected increased inducible NOS expression in the plaques of mice exposed to PM2.5 in both high-fat and normal chow groups [13], which is consistent with our results.

It is not clear yet how body weight was reduced by PM2.5. Previously, many studies demonstrated that chronic exposure to PM2.5 can induce insulin resistance and metabolic syndrome [46–48]. PM2.5 can also induce systemic inflammation by elevating the circulating TNF-α and IL-6 [49]. In our model, we examined the circulating inflammatory cytokines (TNF-α and IL-6), which were consistent with the previous study. We therefore suggest that chronic exposure to PM2.5 can upregulate the circulating inflammatory cytokines and induce insulin resistance. Further study is needed to demonstrate whether body weight loss is associated with insulin resistance and diabetes.

In the present study, we found that 12-week PM2.5 exposure can increase vascular inflammatory factors, thus induce vascular endothelium dysfunction and intracranial atherosclerosis. Many *in vitro* studies have indicated that PM2.5 exposure can decrease cell viability [30, 50]. Also, PM2.5 exposure downregulated the expression of HDL. Other studies showed that PM2.5 could decrease the antioxidant enzymes SOD and CAT [51]. In our future study, we will compare the effects of PM2.5 on both cell viability in neural cell cultures and cell injury/death in brain tissue. We will also determine what molecules are downregulated by PM2.5, and expand our efforts to confirm the mechanisms underlying the detrimental effects of PM2.5 and beneficial effects of omega-3 fatty acids.

In summary, our data shows that PM2.5 exposure for 12 weeks caused ICA, especially with HCD. This effect appears to be mediated by inducing brain vascular inflammation and endothelium dysfunction. In addition, long-term O3FA dietary supplementation prevented the development of intracranial atherosclerosis.

## MATERIALS AND METHODS

### Animal model

The animal protocol was approved by the Animal Care and Use Committee of Capital Medical University and was consistent with the NIH Guide for the Care and Use of Laboratory Animals. Six-week old male Sprague-Dawley rats were purchased from Charles River Laboratories and randomly grouped into PM2.5 (*n* = 54) or filtered air (FA) group (*n* = 54), with each group further divided into normal chow diet (NCD) (*n* = 18) and HCD with O3FA (*n* = 18) or without (*n* = 18) O3FA treatment. The normal chow group was placed on a maintenance diet, while the high-cholesterol group was fed a daily 1% cholesterol diet for up to 6 or 12 weeks. This cholesterol diet is similar to what has been given in other studies on atherosclerosis in rats [52, 53]. During the first two weeks, L-NAME (3 mg/ml) was added to the high-cholesterol group’s drinking water to induce intimal changes, making the rats susceptible to atherosclerosis since nitric oxide (NO) in the blood vessels prevent vascular inflammation [52, 53]. A total of 600 mg L-NAME per rat was administered over two weeks as supplementation.

### Exposure to PM2.5

Animals were exposed to PM2.5 or FA using a “real-world” PM2.5 exposure system modified from “versatile aerosol concentration enrichment system” (VACES) developed by Sioutas [54] and modified by Chen and Nadziejko [14]. The rats were exposed to PM2.5 or FA for a total of 6 (from August 9, 2016 to September 19, 2016) or 12 weeks (from August 9, 2016 to October 31, 2016). The control (FA) rats in the experiment were exposed to an identical protocol with the exception of a high efficiency particulate-air filter positioned in the inlet valve position to remove all of the PM2.5 in the filtered air stream. The rats in the exposure chamber were fed commercial mouse chow and distilled water, and were housed under controlled temperature (22 ± 2°C) and relative humidity (40–60%) conditions with a 12 h light/dark cycle. On the final day of the exposure, all rats were euthanized and tissue samples were collected for further studies.

### O3FA administration

The O3FA treatment group received supplementation of O3FA (5 mg/kg/per day) by oral gavages. O3FA (62160 Sigma-Aldrich, St. Louis, MO, USA) is called α-Lnn, cis,cis,cis-9,12,15-Octadecatrienoic acid, and serves as a precursor to eicosapentaenoic acid (EPA) but not to docosahexaenoic acid (DHA). DHA is formed from EPA. The O3FA polyunsaturated fatty acids are cis-5,8,11,14,17-EPA and cis-4,7,10,13,16,19-DHA.

### Body weight and blood lipids

During the exposure, body weight was recorded every week. To evaluate the impact of inhalation exposure to PM2.5 on lipid homeostasis in animals fed with NCD or HCD, we examined lipid profiles with the blood samples of rats exposed to PM2.5 or FA for 6 or 12 weeks. Systemic blood samples (3.0 ml) were collected before animal sacrifice for the determination of triglycerides (TG), high-density lipoprotein (HDL), low-density lipoprotein (LDL), and cholesterol (CHO) at 6 or 12 weeks. Blood was centrifuged at 2000 r/min, and the plasma was taken for analysis with an automatic biochemistry analyzer (COBAS INTEGRA800).

### Vessel morphometry

Animals were deeply anesthetized with Nembutal (60 mg/kg, i.p.) and sacrificed by cardiac perfusion with saline followed by 4% paraformaldehyde in 0.1 M phosphate buffer (PB) at pH 7.4. Morphometric atherosclerosis in the MCA at 6 or 12 weeks was determined by the ratio of the vessel’s lumen to its wall area. Transverse 6 µm-thick sections of the MCA, cut at the level just above the inferior horn of the lateral ventricles, were stained with hematoxylin-eosin [55]. Two consecutive sections from each animal were measured in both hemispheres with the assistance of image analysis software. Lumen-to-wall ratio from three to five levels of MCA per section was averaged in each animal. All measurements were made in a blinded manner.

### Isolation of brain microvessels

As described previously by us [28], after cardiac perfusion with saline as described above, brains were removed and homogenized in 3 vol. ice-cold sucrose buffer with a Dounce homogenizer provided with a tightly fitting pestle, followed by centrifugation at 4°C for 10 min at 1000 g. After discarding the supernatant, the dense white layer of myelin in the upper part of the pellet was removed and the pellet re-suspended again in 3 vol. of cold sucrose buffer on ice, followed by homogenization and centrifugation at 4°C for 10 min at 1000 g. The sediment was then re-suspended in sucrose buffer and centrifuged twice for 30 s at 100 g. The supernatants were pooled and washed twice with sucrose buffer and once with phosphate-buffered saline+0.1% bovine plasma albumin at 200 g. The final pellet was suspended in 1.0 ml of phosphate-buffered saline+0.1% bovine plasma albumin, centrifuged at 14,000 g and the precipitate was stored at –70°C.

### Real-time PCR

The isolated cerebral microvessels were homogenized and RNA was isolated using Trizol reagent (Invitrogen, Carlsbad, CA) according to the manufacturer’s instructions. Total RNA was then converted into cDNA using the High Capacity cDNA Reverse Transcription Kit (Applied Biosystems, Foster City, CA). The quantification of gene expression was determined by Prism 7500 real-time PCR (Applied Biosystems, CA, USA). All reactions were performed under the following conditions: 95°C for 15 minutes, 40 cycles of 95°C for 10 seconds, and 60°C for 30 seconds. The primers for rat interleukin 6 (IL-6), tumor necrosis factor alpha (TNF-α), monocyte chemoattractant protein-1 (MCP-1), interferon gamma (IFN-γ), vascular cell adhesion molecule 1 (VCAM-1), inducible nitric oxide synthase (iNOS) and β-actin are shown in Table 2. Beta-actin was used as the control gene and all data are represented as relative mRNA expression on gene expression.

**Table 2:** Primers for real-time polymerase chain reaction (PCR) analysis

Genes	Forward Primer (5′–3′)	Reverse Primer (5′–3′)
TNF-α	TACTCCCAGGTTCTCTTCAAGG	GGAGGCTGACTTTCTCCTGGTA
IL-6	GAGTTGTGCAATGGCAATTC	ACTCCAGAAGACCAGAGCAG
iNOS	CTTTCTGGCAGCAGCGGCTC	GCTCCTCGTAAGTTCAGC
VCAM1	TTTGCAAGAAAAGCCAACATGAAAG	TCTCCAACAGTTCAGACGTTAGC
MCP-1	TGAACTTGACCCATAAATCTGAAG	AAGGCATCACATTCCAAATCAC
IFN-γ	ATGGATGCTATGGAAGGAAAGA	GGCACACTCTCTACCCCAGAA
β-actin	ATCGTGGGCCGCCCTAGGCACC	CTCTTTAATGTCACGCACGATTTC

### Western blotting

Cerebral microvessels at 6 or 12 weeks were processed and analyzed with Western blot as described previously by us [28, 56]. Briefly, the isolated brain microvessels as described above were homogenized and processed for Western blotting. Primary antibodies, including anti-TNF-α (1:1000, ab6671, Abcam, Cambridge, MA, USA), anti-IL-6 (1:1000, ab9324, Abcam, Cambridge, MA, USA), anti-MCP-1 (1:2000, ab25124, Abcam, Cambridge, MA, USA), anti-INF-γ (1:1000, ab7740, Abcam, Cambridge, MA, USA), anti-iNOS (1:2000, ab15323, Abcam, Cambridge, MA, USA), anti-VCAM-1 (1:5000, ab134047, Abcam, Cambridge, MA, USA), and anti-β-actin (1:5000, A5060, Sigma-Aldrich, St. Louis, MO, USA), were incubated on the membrane at 4°C overnight. Protein expression was detected using an enhanced chemiluminescence kit (Millipore, Billerica, MA, USA).

### Statistical analyses

Data are expressed as mean ± SE unless otherwise indicated. The differences between the mean values of two groups were determined by Student’s *t*-test. Associations between the different variables were examined by one-way ANOVA, followed by post hoc comparisons using the Tukey’s multiple paired comparison test. All analyses were performed using Graphpad Prism v5.0 (Graphpad Software, San Diego, CA). In all cases, a *p* value of < 0.05 was considered as statistically significant.
